# Drug development for sickle cell disease: repeated setbacks, yet there remains an optimistic outlook for future breakthroughs

**DOI:** 10.1016/j.htct.2025.106251

**Published:** 2026-01-12

**Authors:** Rodolfo Cançado, Fernando Ferreira Costa

**Affiliations:** Faculdade de Ciências Médicas da Santa Casa de São Paulo (FCMSCSP), São Paulo, SP, Brazil; Faculdade de Ciências Médicas da Universidade Estadual de Campinas (FCM-UNICAMP), Campinas, SP, Brazil

To the editor:

Sickle cell disease (SCD) is a chronic and debilitating genetic blood disorder that affects approximately 6.5 to 9 million individuals worldwide. It is estimated that more than 90% of them are located in Africa and India [1,2].

The basic event of the pathophysiology of the disease, namely the polymerization of deoxy HbS, has been recognized for approximately the last fifty years [3]. Since then, we have seen a continuous search for drugs that can interfere with and reduce hemoglobin S polymerization as a potential therapy for SCD. Approximately a hundred possible anti-sickling agents have been described. Several of these, with some effects *in vitro*, were tested in several clinical trials without success [3, 4, 5, 6, 7, 8, 9, 10, 11, 12, 13, 14, 15]. More recently, newer molecular biology methods and animal models of SCD have helped characterize the complex chain of events, beyond Hb polymerization, responsible for most of the acute and chronic organ lesions of SCD. Thus, several other essential steps responsible for the vaso-occlusion and organ damage have been discovered, such as endothelial cell lesions, the critical role of hemolysis in inflammation and organ damage, increased adhesion of red and white blood cells and platelets to the endothelium, sterile inflammation, reduced nitric oxide (NO) levels, activation of thrombosis and lesions secondary to oxidation [2]. These new data resulted in a new wave of possible new treatment targets, which started the search for new of families of molecules.

However, until now, the only molecule that has been able to reduce the polymerization of deoxy HbS in humans with confirmed clinical effects is hydroxyurea (HU). The predominant effect of HU is to increase the production of HbF, a hemoglobin well known as a molecule that can reduce the desoxy HbS polymerization. Moreover, HU presents other actions, such as, for example, reducing white and red blood cell adhesion to the endothelium, acting as a NO donor, and reducing inflammation secondary to hemolysis. HU was approved by the FDA for treatment of adults with SCD in 1998 and for children in 2017. It is effective and safe for all ages and in all regions of the planet, and there is robust evidence that treatment with HU reduces the number of vaso-occlusive crises and acute chest syndrome, increases patient survival, and decreases chronic organ damage. Although there is a percentage of patients that do not respond to the treatment and there are several collateral effects that should be monitored, there are no other drugs that have shown clinical effects comparable to those of HU. In fact, HU should be considered, today, the standard drug for SCD and universal access to this drug should be a priority, particularly for patients in countries with low and medium incomes (LMIC), such as regions of Sub-Saharan Africa and India [2,15].

In addition, several basic procedures should be recognized as essential to increase the survival of SCD patients, irrespective of any specific drugs for blocking polymerization. Solid and well known data have shown that early universal diagnosis of the disease (as achieved by newborn screening), well organized infrastructure and health care for children with SCD, procedures to prevent complications such as pneumococcus, Haemophiles Influenzae and meningococcal vaccines, prophylactic penicillin, transcranial doppler (TCD), adequate and well controlled transfusion programs, adequate patient registries, well-trained health providers, among others, can significantly increase the survival of patients, as has been seen in some high income countries such the US, UK and France. Unfortunately, in most low-middle income countries (LMICs), even basic procedures are not available, and the implementation of achievable goals aimed at reducing the mortality and morbidity associated with this long-neglected disease is urgent [2,16].

Despite all of the benefits derived from the treatment with HU and other well-consolidated measures, it is recognized that there are several unmet needs in the treatment of SCD and new treatments that target the underlying course of SCD, as well as its acute and chronic complications are necessary. Regrettably, with exception of HU, in the past few decades there has been an unfortunate failure to provide new disease-modifying treatments, due to a lack of benefit or potential harm, as demonstrated by clinical trials [2,4,17, 18, 19, 20]. Remarkably in the last eight years, three new drugs were approved by the US control agency, the FDA, including L-glutamine, crizanlizumab and voxelotor. The very disturbing fact is that two of them (crizanlizumab and voxelotor) were removed from the market due to lack of benefit or emerging safety concerns and re-evaluation of their risk-benefit profiles [17,18,20, 21, 22].

In September 2024, Pfizer voluntarily withdrew voxelotor, an anti-sickling agent for SCD, from the global market based on emerging clinical data indicating that the drug's risks outweigh its benefits in SCD management.3 Studies have indicated an elevated risk of vaso-occlusive crises and fatalities among individuals receiving voxelotor treatment. In a multicenter trial (NCT04218084) involving 236 children with sickle cell disease (SCD) who were at high risk of stroke, there were eight deaths in the voxelotor group, compared to two in the placebo group, prompting an early termination of the trial [23].

Another trial (NCT05561140) focused on adolescents and adults with SCD and leg ulcers, and reported eight deaths among 88 patients treated with voxelotor during the open-label phase [24]. These outcomes led Pfizer to discontinue the distribution, recall existing stock, and cease all ongoing clinical trials involving voxelotor.

Given the probable efficacy, benefits and favorable safety profile from the pivotal phase 2 SUSTAIN study (NCT01895361), along with pharmacokinetic and pharmacodynamic evaluations of the dosing regimen, crizanlizumab, an anti-adhesive monoclonal antibody, was seen as a potential disease-modifying therapy for reducing vaso-occlusive crises (VOCs) in SCD [25]. It received conditional approval from the regulatory agencies in the USA, Europe and Brazil. Unfortunately, the primary results from the Phase 3 STAND trial (NCT03814746) indicated no significant difference between the crizanlizumab groups (5.0 mg/kg and 7.5 mg/kg) and the placebo group in the annualized rate of vaso-occlusive crises (VOC) that necessitated a healthcare visit or home management for patients with sickle cell disease (SCD) aged 12 years and older. The safety profile of crizanlizumab was consistent with previous studies, and no new safety issues were identified with either dose. Although the drug has been withdrawn from the market, it remains available through compassionate use and other programs in several regions.

Despite these adverse results, the game seems not to be over for crizanlizumab. New data from clinical trials involving different regimens and age groups of SCD patients continue to emerge about crizanlizumab. This data reinforces the novel drug’s safety and tolerability globally. The subsequent phase 2 open-label SOLACE-adults study analyzed long-term (i.e., ≥3 years) follow-up on the effects of crizanlizumab (5.0 mg/kg and 7.5 mg/kg) in adult patients with SCD (N=57) [26]. Similarly, the Phase 2 SOLACE-kids study examined children with SCD aged 12 to under 18 years (N=53) [18]. Both studies showed reductions from baseline in VOC-related healthcare visits, hospitalizations, emergency room visits, and a consistent safety profile for crizanlizumab. Crizanlizumab was made available through a managed access program (MAP, NCT03720626). This analysis assessed the effects of 12 months of crizanlizumab treatment on vaso-occlusive crises (VOCs) and opioid use for managing VOC-related pain in patients with sickle cell disease (SCD) participating in the MAP. Crizanlizumab showed promise in reducing VOC incidents, regardless of SCD genotype or previous hydroxyurea treatment, and in decreasing opioid consumption. The safety profile of crizanlizumab aligned to prior findings [27].

To test the effectiveness of crizanlizumab outside a pandemic context, Novartis is conducting the CSEG101A2303 study (SPARKLE, NCT06439082). This phase III, multi-center, randomized, placebo-controlled, double-blind trial is designed to evaluate the efficacy and safety of crizanlizumab (5 mg/kg) compared to a placebo, with or without hydroxyurea/hydroxycarbamide treatment, in patients with sickle cell disease (SCD) aged 12 and older who experience frequent vaso-occlusive crises (4-12 events in the 12 months leading up to the screening visit). Participants will be randomly assigned in a 2:1 ratio to receive either crizanlizumab 5 mg/kg or a placebo. Central randomization will be stratified based on concurrent use of HU/HC (yes/no) and region (South America, North America, and sub-Saharan Africa) at baseline [28].

On August 15, 2025, Pfizer Inc. announced results from the Phase 3 THRIVE-131 (NCT04935879) trial evaluating inclacumab, an investigational P-selectin inhibitor and has decided to terminate the study [29]. The study enrolled 241 participants with SCD, 16 years of age and older. Inclacumab was generally well-tolerated, but the study did not meet its primary endpoint of a significant reduction in the frequency of VOCs in participants administered inclacumab compared to those given a placebo every 12 weeks over 48 weeks [29].

It is very difficult to describe all the causes of this repetitive failure to find new drugs with significant clinical effects. Moreover, the approval and subsequent removal from the market of Voxelotor and Crizanlizumab sparked an intense debate among researchers and physicians working with SCD [19,20, 21, 22]. Despite all these problems, important lessons emerge from this disappointing scenario of failure in developing new drugs for treating SCD and are described below [19,21,22]:

○ The pathophysiology of SCD turned out to be very complex with multiple pathways.
○ Geographic variability remains a significant challenge while evaluating real-world data on novel SCD therapies. SCD outcomes vary widely across different countries and continents. Recruitment across diverse geographical areas should consider patients’ demographics, including various regions (e.g., Africa, Brazil and India) and ethnicities. Although the disease had similar manifestations across ethnicities, variations in the use of health-care facilities, socio-cultural factors, and management of pain across different regions could have marked differences in its results [15,21].
○ The complex interplay between environmental and genetic factors, trial settings, healthcare usage and practices, and how they influence treatment efficacy.
○ It’s time to reach a consensus on the definitions of VOC in patients who receive these therapies. While clinical trials of therapies for patients with SCD often rely on the reduction or elimination of VOC pain events as a clinical endpoint, definitions of what constitutes a VOC pain event differ between trials and have no impact on efficacy assessments between clinical trials [30,31].
○ A possible premature approval of drugs by authorities, their promotion by healthcare professionals and patient associations, and the pursuit by pharmaceutical companies to access the market rapidly should be rethought and better discussed [15,19,21,22].

What suggestions can be considered to counteract this unsatisfactory situation, effectively reduce health disparities, and promote the global advancement of efficient and accessible therapies? [4,19]. As already mentioned, there are well-known and recognized measures in the comprehensive management and patient-focused care of SCD that are essential. Among these, it is essential to emphasize universal newborn screening, providing access to specialized healthcare for all patients with SCD, regardless of their location, making HU accessible and affordable, and improving the availability and safety of blood transfusions. In addition, there are several other actions that should be considered [2,15,19,21,22]:

1. Improving clinical trial designs: focus on meaningful clinical outcomes, incorporate diverse patient populations, always include patient participation, and consider variations in SCD outcomes between different countries and continents [30,31].
2. Real-world evidence should be considered as the primary reference for translating clinical trial results to achieve and reliability across various patient groups.
3. Innovative therapeutic approaches: explore personalized therapies based on combined treatments that target various pathophysiological pathways in SCD, such as combination therapies, which are currently underexplored.
4. Collaboration and communication involve fostering dialogue among experts, patients, funding bodies, universities, pharmaceutical companies, regulatory authorities, and other stakeholders.
5. Increasing research funding requires promoting a more effective blend of academic and industry-driven studies, engaging patients from the outset, and conducting more robust clinical trials with suitable patient populations and endpoints. [32,33]

As of now, hydroxyurea remains the only approved disease-modifying therapy for sickle cell disease (SCD). Given the significance of SCD as a global public health issue, it is imperative to develop new therapeutic options. Encouragingly, emerging drugs - such as pyruvate kinase activators and those that either inhibit the polymerization of sickle hemoglobin or enhance the production of hemoglobin F - are advancing to late-stage clinical trials [21,22]. Substantial progress is being achieved in so-called curative therapies, including bone marrow transplantation and gene therapy. The possibility of doing HSCT with haploidentical donors seems to be a major improvement [34]. Two gene therapy procedures are now approved by the FDA (gene editing and gene addition), but these procedures are extremely expensive, making access impossible for most patients with SCD [35].

It is crucial to emphasize the need for transparent and careful communication when approving or withdrawing therapies for SCD [36]. When new medications are approved, it is essential to proceed cautiously, clarifying known toxicities, uncertainties, and the provisional nature of the approvals, avoiding exaggerated promises. There must be long-term post-approval, real-world outcomes, supported by multiple data registries, to ensure that subtle problems and other benefits can be identified [22]. Obviously, it is essential to ensure informed consent from all the patients. In the case of medication withdrawals, it is very important to provide the data that justifies the decision promptly [36].

## Conflicts of interest

The authors declare no conflicts of interest.
